# Initial insights into the impact and implementation of Creating Active Schools in Bradford, UK

**DOI:** 10.1186/s12966-023-01485-3

**Published:** 2023-07-05

**Authors:** Jade L. Morris, Anna E. Chalkley, Zoe E. Helme, Oliver Timms, Emma Young, Gabriella M. McLoughlin, John B. Bartholomew, Andy Daly-Smith

**Affiliations:** 1 Faculty of Life Sciences and Health Studies, University of Bradford, Richmond Road, Bradford, UK; 2 Centre for Applied Education Research, Wolfson Centre for Applied Health Research, Bradford Royal Infirmary, Bradford, West Yorkshire UK; 3 Centre for Physically Active Learning, Faculty of Education, Arts and Sports, Western Norway University of Applied Sciences, Sogndal, Norway; 4 Reducing Inequalities in Communities schools project, Public Health, Department of Health & Wellbeing, City of Bradford Metropolitan District Council, Bradford, UK; 5 College of Public Health, Temple University, Philadelphia, USA; 6 Implementation Science Center for Cancer Control and Prevention Research Center, Brown School, Washington University in St. Louis, St. Louis, USA; 7 Department of Kinesiology and Health Education, The University of Texas at Austin, Austin, TX USA

**Keywords:** Creating Active Schools, Implementation science, Whole-school physical activity, Physical activity promotion, Children, Implementation outcomes, Implementation determinants

## Abstract

**Background:**

Few whole-school physical activity programmes integrate implementation science frameworks within the design, delivery, and evaluation. As a result, knowledge of the key factors that support implementation at scale is lacking. The Creating Active Schools (CAS) programme was co-designed and is underpinned by the Capability, Opportunity, Motivation and Behaviour (COM-B) model and the Consolidated Framework for Implementation Research (CFIR). The study aims to understand the initial impact and implementation of CAS in Bradford over 9 months using McKay’s et al.’s (2019) implementation evaluation roadmap.

**Methods:**

Focus groups and interviews were conducted with school staff (n = 30, schools = 25), CAS Champions (n = 9), and the CAS strategic lead (n = 1). Qualitative data were analysed both inductively and deductively. The deductive analysis involved coding data into *a priori* themes based on McKay et al’s implementation evaluation roadmap, using a codebook approach to thematic analysis. The inductive analysis included producing initial codes and reviewing themes before finalising.

**Results:**

Identified themes aligned into three categories: (i) key ingredients for successful adoption and implementation of CAS, (ii) CAS implementation: challenges and solutions, and (iv) the perceived effectiveness of CAS at the school level. This included the willingness of schools to adopt and implement whole-school approaches when they are perceived as high quality and aligned with current school values. The programme implementation processes were seen as supportive; schools identified and valued the step-change approach to implementing CAS long-term. Formal and informal communities of practice provided “safe spaces” for cross-school support. Conversely, challenges persisted with gaining broader reach within schools, school staff’s self-competence and shifting school culture around physical activity. This resulted in varied uptake between and within schools.

**Conclusions:**

This study provides novel insights into the implementation of CAS, with outcomes aligning to the adoption, reach, and sustainability. Successful implementation of CAS was underpinned by determinants including acceptability, intervention complexity, school culture and school stakeholders’ perceived self-efficacy. The combination of McKay’s evaluation roadmap and CFIR establishes a rigorous approach for evaluating activity promotion programmes underpinned by behavioural and implementation science. Resultantly this study offers originality and progression in understanding the implementation and effectiveness of whole-school approaches to physical activity.

**Supplementary Information:**

The online version contains supplementary material available at 10.1186/s12966-023-01485-3.

## Background

Children’s physical activity levels remain consistently low, with 60% not meeting global recommendations [1–3]. Deepening health inequality, levels of inactivity are higher in areas of high deprivation and diverse ethnic minority communities - especially of South Asian heritage [4, 5]. Schools are seen as a key setting within which to intervene to increase children’s physical activity levels [6]. Yet, recent review-level evidence suggests that previous school-based physical activity interventions only achieved small, unsustained improvements [7–9]. Of further concern, positive effects observed in efficacy and effectiveness trials often diminish - known as a voltage drop [10] - as programmes scale for broader implementation and effectiveness [11, 12].

UK and international guidance promotes whole-school approaches as a “best investment” to tackle childhood inactivity due to engaging a large number of diverse children over an extended period [13–16]. However, there is a lack of understanding of what constitutes a whole-school physical activity approach [17], programmes failing to recognise schools as complex adaptive systems [17] and taking a context specific approach [18], limited multi-stakeholder input in programme design [19] and poor use of implementation theory within programme design, delivery and evaluation [20]. Resultantly, programmes often focus on providing single or multi-component opportunities for children to be physically active [21, 22], and fail to address the higher-level systems issues such as policy, environments and the role of different stakeholder groups. Integrating implementation science frameworks within the development of whole-school approaches reinforces the importance of moving beyond addressing school stakeholders’ delivery of physical activity to also consider higher-level system factors [23].

The Creating Active Schools (CAS) framework [17] responded to calls for the co-production of school-based activity promotion programmes [19] by involving nine stakeholder groups in the initial design process. The framework informed the rigorous development of the CAS programme, embedding behavioural [24] and implementation science [25, 26] in the design, delivery and evaluation of a programme focussed on organisational and cultural change for physical activity in schools [27]. The CAS programme is underpinned by the Consolidated Framework for Implementation Research (CFIR), a conceptual framework that organises implementation domains across five domains: (i) characteristics of individuals (school stakeholders), (ii) inner setting (schools), (iii) outer setting (beyond the school), (iv) intervention characteristics, and (v) implementation processes [25]. Despite preliminary evidence on the effectiveness of CAS [27], little is known about the determinants that influence initial implementation. Moreover, while CFIR is widely adopted in healthcare research [28–31], there has only been a limited use within whole-school physical activity and wellness programmes [32, 33].

This study was designed to address these issues through an evaluation of CAS implementation, guided by the recent implementation evaluation roadmap [34] which provides a minimum data set of indicators to rigorously evaluate school-based physical activity programme implementation [26, 34]. The framework establishes a “common language” for five implementation outcomes – defined as the effects of deliberate actions to implement and scale up an intervention [35] – and 10 implementation determinants – referring to the range of contextual factors that influence implementation and scale-up in schools [36]. Evaluating CAS implementation using a rigorous framework provides a valuable scientific contribution to the implementation science field related to whole-school physical activity; with a specific contribution to schools in deprived communities. The study aims to understand the initial impact and implementation of CAS and to address three key objectives:

1. To establish the implementation processes and practices in schools that have adopted CAS.
2. To identify the key innovation characteristics of CAS that are related to successful school adoption and implementation outcomes.
3. To assess initial perceptions of CAS and its impact on enhancing policy, stakeholder behaviour, environments, and opportunities to facilitate whole-school physical activity provision.

## Methods

### Intervention

**Fig. 1:**
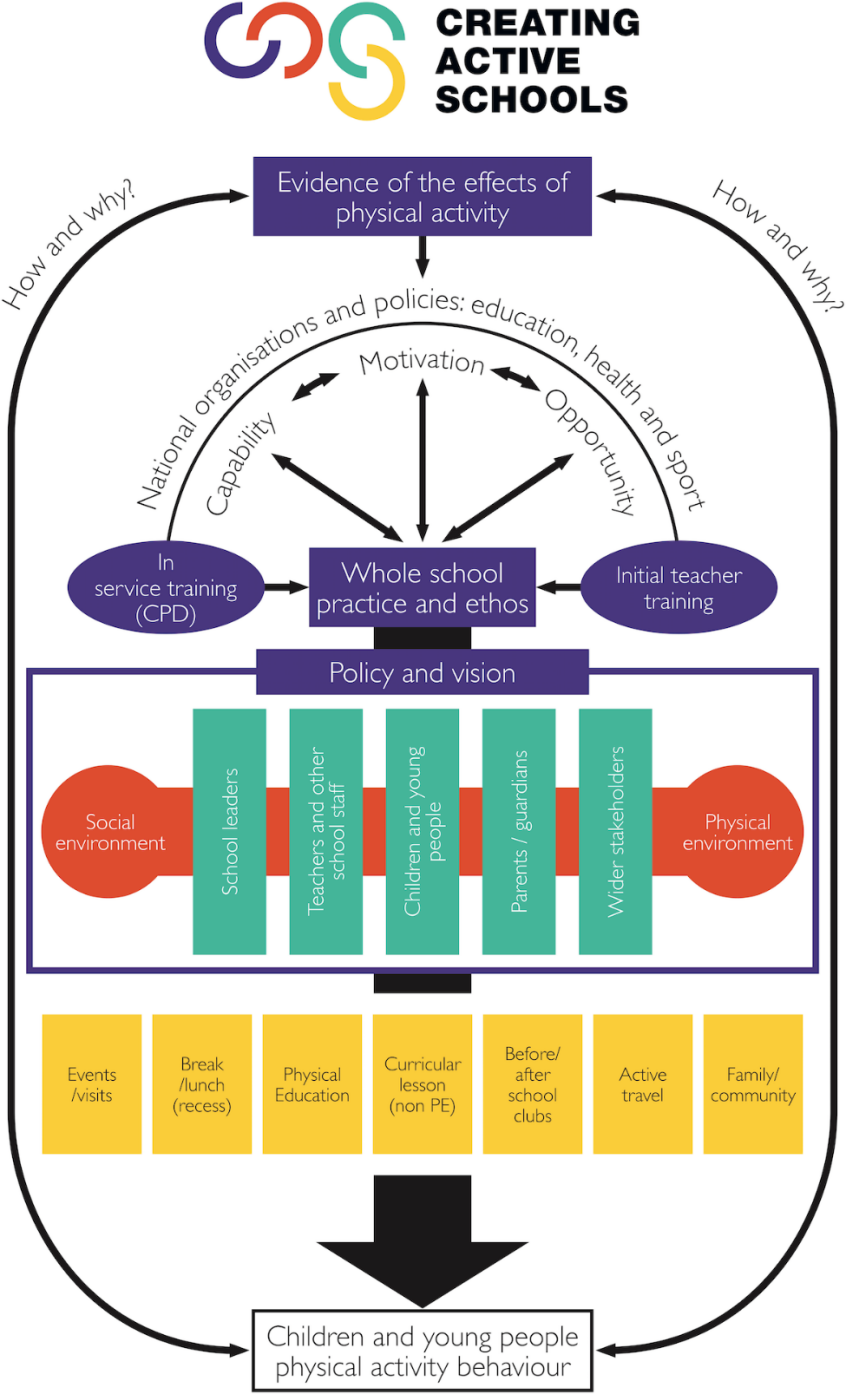
Creating Active Schools Framework

**Fig. 2:**
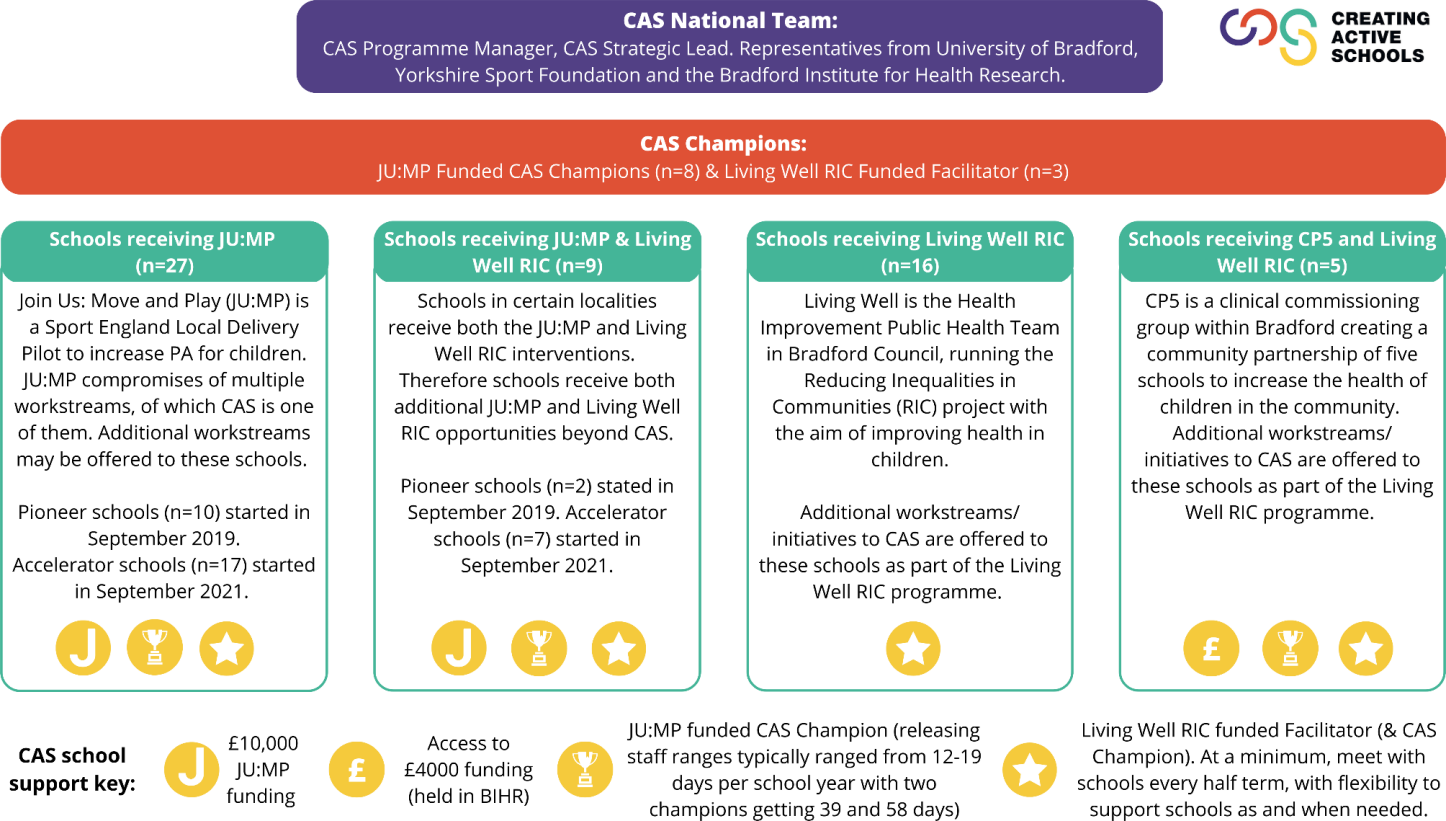
CAS Delivery Model in Bradford

CAS programme implementation in Bradford began in September 2021. The programme focuses on schools assets (e.g., facilities, environments, staff, capacity) to promote behaviour change using the COM-B framework [24] across four areas: (i) policy, (ii) environments, (iii) stakeholders, and (iv) opportunities (Fig. 1). For further programme details refer to Helme et al., [27]. The programme was developed by a CAS strategic lead based in Bradford with the continuous support of the CAS national team (Yorkshire Sport Foundation, University of Bradford, and Bradford Institute for Health Research). Schools in Bradford (n = 57) were recruited through three delivery models, all of which provided different amenities and funding opportunities (see Fig. 2).

The CAS national team recruited locality leads *(CAS Champions; n = 11)* from local schools and public health teams to support schools to implement CAS. They onboarded schools to the programme and supported them through the four-stage CAS process, trained in-school CAS leads and developed inter-school communities of practice (CoP). CAS Champions received two days of training provided by the CAS national team and regular support via webinars and face-to-face meetings with the Bradford CAS strategic lead throughout the year.

Once recruited, schools took part in the four-stage annual CAS cycle (see 27). In brief, this included (i) in-school CAS leads completing the profiling tool to assess current whole-school physical activity provision, (ii) in-school CAS leads completing a Planning for Change document using the APEASE quality assurance criteria [37] to identify evidence-informed initiatives, (iii) CAS Champions supporting the implementation of individual and collective school initiatives, and (iv) schools evaluating initial impact to inform the next annual cycle. Further school-based support was provided via termly formal CoP for all schools, which included networking opportunities, sharing case studies and bespoke professional development.

### Design

This study used a qualitative descriptive design [38] involving semi-structured focus groups and interviews to gather contextually rich data and understand the diversity of experiences associated with CAS participation. Ethical clearance was granted by the University of Bradford Research and Innovation Services (reference = E926).

### Selection and recruitment

To gain multiple perspectives on CAS implementation in Bradford, recruitment took part in two phases. Phase one included inviting in-school CAS leads, teachers, and members of school senior leadership teams (SLT) that were attending the 2022 summer CoP (June 2022; n = 25). Phase two involved inviting the CAS Strategic Lead (n = 1), Living Well CAS Champions (n = 3) and Join Us Move Play (JU:MP) CAS Champions (n = 8) who were attending the CAS Champion summer training day (July 2022). Written consent was gained before any focus groups commenced.

### Data collection

Phase one took place during the 2022 CAS Summer CoP, whereby six focus groups were conducted with attending school staff (n = 30) from 25 (out of 57) Bradford-based CAS schools. Phase two took place during the CAS Champion training day (July 2022), whereby three focus groups took place with attending CAS Champions (n = 9). In addition, a semi-structured interview was conducted with the CAS strategic lead. One of three authors led the focus groups and the interview (JLM, AC, ZH). No prior relationships existed between interviewers and interviewees. All discussions were audio recorded and lasted between 36 and 67 min (M = 53 min).

The semi-structured interview and focus group guides were ordered into three sections: (i) adoption and attrition, (ii) effectiveness, and (iii) implementation (supplementary file 1). Recordings were transcribed verbatim by the first author and re-checked for accuracy before anonymisation.

### Data analysis

Analysis incorporated both a data-driven inductive approach using codebook thematic analysis [39] and a deductive approach guided by *a priori* themes based on McKay et al’s [34] implementation evaluation roadmap. We amended the adoption construct as the definition in McKay et al’s [34] did not align with the CAS programme (see Table 1).

**﻿Table 1﻿:** Overarching themes, descriptions, and subthemes

Themes	Theme description	Sub-themes
***Implementation outcomes***		
Adoption ^1^	The initial decision to onboard and adopt CAS ^*^	Attractiveness of the CAS ethos ^1.1^
		Existing commitment to a physical activity agenda though locality-based projects ^1.2^
		Initial financial incentive ^1.3^
		School implementation readiness ^1.4^
Dose delivered ^2^	Intended CAS components delivered by delivery team	CAS Champions facilitating delivery ^2.1^
Reach ^3^	Proportion of the intended priority audience (schools and school staff) participating in CAS.	Limited visibility & permeation of CAS in schools ^3.1^
		Identified approaches to increase reach of CAS ^3.2^
Fidelity ^4^	The extent to which CAS is implemented as prescribed in the intervention protocol - by the delivery team	CAS Champions to act as friendly critics to schools ^4.1^
Sustainability ^5^	Whether CAS continues to be delivered and/or individual behaviour change is maintained	Implementation efforts to ensure sustainability ^5.1^
		CAS Champion support a necessity for sustainability ^5.2^
***Implementation determinants***		
Context ^6^	Aspects of the larger social, political, & economic environment that may influence CAS implementation	Opportunities and challenges provided by Covid-19 ^6.1^
		Impact of financial differences between schools ^6.2^
		Added value of CAS to other locality initiatives addressing health inequalities in Bradford ^6.3^
		Ofsted priorities misalign with CAS ^6.4^
		Staff turnover implications on CAS implementation ^6.5^
		Limited opportunity for CAS Champion training ^6.6^
Acceptability ^7^	Perceptions among the delivery team that CAS is agreeable, palatable, or satisfactory	Initial enthusiasm and anticipation for CAS ^7.1^
		CAS Champion support promotes wider school buy-in ^7.2^
Adaptability ^8^	Extent to which CAS can be adapted, tailored, refined, or reinvented to meet local needs	Simplification of CAS tools to increase useability ^8.1^
		Flexibility in the school delivery model ^8.2^
Feasibility ^9^	Perceptions among the delivery team that CAS can be successfully used or carried out within school/s	Staff’s (limited) Capacity ^9.1^
		In-school CAS lead’s autonomy to make decisions ^9.2^
Compatibility (appropriateness) ^10^	Extent to which CAS fits with the mission, priorities, and values of schools	CAS meeting an identified need in school ^10.1^
		School’s see value of CAS ^10.2^
		Incompatibility and less perceived value of CAS ^10.3^
Cost ^11^	Money spent on design, adaptation, and implementation of CAS	Financial and opportunity costs of releasing CAS Champion ^11.1^
Culture ^12^	Schools’ norms, values, & basic assumptions around selected health outcomes (physical activity)	Recognition for whole-school culture around physical activity ^12.1^
		Idealism of school physical activity policy (currently missing in schools) ^12.2^
		SLT support of CAS required to leverage whole-school buy-in ^12.3^
		How CAS is operationalised in school (endemic top-down school approach) ^12.4^
		CAS seen as synonymous with PE & Sport ^12.5^
		Challenges to staff buy-in ^12.6^
		Changes to staff’s mindset since adopting CAS ^12.7^
Dose (satisfaction) ^13^	Delivery team’s satisfaction with CAS (and encompassing components) and with interactions with the support system	General satisfaction with CAS ^13.1^
		CAS Champions and facilitator ^13.2^
		CAS communities of practice facilitating networking opportunities ^13.3^
		Administrative tasks (e.g. profiling tool) seen as laborious but generally beneficial ^13.4^
		JU:MP related satisfaction ^13.5^
Complexity ^14^	Perceptions among the delivery team that CAS is relatively difficult to understand and use; number of different intervention components	Multiple health-based projects in Bradford causing perplexity ^14.1^
		Initial bewilderment alleviated over time ^14.2^
		CAS Champion support increasing clarity for school staff ^14.3^
Self-efficacy ^15^	Delivery team’s belief in its ability to execute courses of action to achieve implementation goals	Development of school staff’s confidence ^15.1^
		CAS Champions differing capabilities ^15.2^
Perceived effectiveness ^16^	Anecdotal effectiveness on whole-school physical activity aligning to the CAS framework (PESO)	Positive changes to school policy ^16.1^
		Positive changes to the school environment ^16.2^
		Positive changes to school-based stakeholders ^16.3^
		Increases in physical activity opportunities ^16.4^
		Perceived impact on children’s physical activity levels ^16.5^

*Note.* * The description for adoption comes from Proctor et al. [35] instead of the implementation evaluation roadmap [34]. Superscript numbers against themes and sub-themes are used to guide the reader to see alignment through the text

Once transcribed, transcripts were read and reread to become familiar with the breadth and depth of the data and to generate preliminary coding ideas and notes. A coding template was generated in Microsoft Excel with rows representing *a priori* themes. JLM led the analysis, supported by AC, OT, and EY. Data were copied into the matrix from transcripts and referenced, allowing the source to be traced and the process to be examined and replicated. Analytic memos were produced to capture impressions and early interpretations. If new codes were proposed that fell outside of, or disagreed with, the agreed coding frame, then they were revised by the four researchers and transcripts were re-read considering the new structure. Codes were collected under the *a priori* themes before comparing coding clusters together. Coding was hierarchical with variation in a given theme being coded under sub-themes. For example, within the ‘Complexity’ theme, we identified the sub-themes of ‘Multiple health-based projects in Bradford causing perplexity’, ‘Initial bewilderment alleviated over time’ and ‘CAS Champion support increasing clarity for school staff’. Trustworthiness was maximised by conducting checks of agreement in interpretation through double coding (AC, OT, EY) and verifying different coders found relevant text using code descriptions. Interpretations were openly discussed and challenged by critically probing for explanations to achieve a final consensus. Additionally, the inclusion of supporting quotations from the range of participants that were interviewed enhanced trustworthiness [40].

## Results

Participants consisted of a CAS strategic lead, CAS champions, SLTs, teachers, and wider school staff (see Table 2). Twenty-two of the 30 school stakeholders adopted the role of ‘in-school CAS lead’. Of which, 14 (64%) were a Physical Education (PE) lead (and a class teacher), PE teacher or coach, four (18%) were class teachers, three (14%) were SLT and one (4%) was also a Special Educational Needs and Disabilities (SEND) lead.

**Table 2:** Summary of participants by stakeholder group

Participant	Gender	CAS Role	School Role
P1	Male	CAS Strategic Lead	N/A
P2	Female	CAS Champion & Living Well RIC Facilitator	N/A
P3	Male	CAS Champion & Living Well RIC Facilitator	N/A
P4*	Male	CAS Champion	PE Lead
P5	Male	CAS Champion	PE Lead
P6	Male	CAS Champion	Teacher & PE Lead
P7	Male	CAS Champion	PE Lead
P8*	Male	CAS Champion	PE Teacher
P9*	Female	CAS Champion	Teacher & PE Lead
P10	Female	CAS Champion	Secondary School PE Teacher
P11	Male	In-school CAS Lead	Senior Leadership Team
P12	Male	In-school CAS Lead	Teacher
P13	Female	In-school CAS Lead	Teacher & PE Lead
P14	Male	In-school CAS Lead	Teacher & PE Lead
P15	Female		Teaching Assistant
P16	Male	In-school CAS Lead	PE Lead
P17*	Male	In-school CAS Lead	PE Lead
P18	Male	In-school CAS Lead	PE Lead
P19	Male	In-school CAS Lead	PE Lead
P20	Female		PE Lead
P21	Male	In-school CAS Lead	Teacher
P22	Female	In-school CAS Lead	Teacher & PE Lead
P23	Female	In-school CAS Lead	Senior Leadership Team
P24	Female	In-school CAS Lead	Teacher
P25	Female		Teacher
P26	Male	In-school CAS Lead	Senior Leadership Team
P27	Male		Senior Leadership Team
P28*	Female	In-school CAS Lead	Teacher
P29	Female		Senior Leadership Team
P30	Female	In-school CAS Lead	SEND Lead
P31	Female		Family Liaison Officer
P32	Female		Senior Leadership Team
P33*	Male	In-school CAS Lead	PE Teacher
P34	Male	In-school CAS Lead	Teacher & PE Lead
P35	Female		Senior Leadership Team
P36	Female	In-school CAS Lead	Teacher & PE Lead
P37	Female	In-school CAS Lead	Teacher & PE Lead
P38	Female	In-school CAS Lead	Teacher & PE Lead
P39	Male	In-school CAS Lead	Teacher & PE Lead
P40	Male	In-school CAS Lead	PE & Wellbeing Coach

*Note.* * Denotes individuals that took part in two focus groups. Once as a CAS Champion (P4, P8, P9) in the champion focus groups and once as an in-school CAS lead (P17, P28, P33) in the school stakeholder focus groups. SEND: Special educational needs and disabilities

Data were coded across all of the 15 *a priori* themes. One additional theme was identified around the perceived effectiveness of CAS (Table 1). Due to the magnitude of the data, we decided to not include sub-themes that were specifically related to the geographical context of the Bradford locality and which fell outside of the scope of this study. A more comprehensive summary of themes, sub-themes, example quotes, and alignment with CFIR is presented in supplementary file 2. Themes are presented below aligning with the study’s objectives: (i) key ingredients for successful adoption and implementation of CAS, (ii) CAS implementation: challenges and solutions (iv) the perceived effectiveness of CAS at the school level. To facilitate understanding, key themes are presented in bold, with sub-themes identified by superscript numbers within the text (Table 1).

### Key ingredients for successful adoption and implementation of CAS

Many school stakeholders referred to the CAS ethos when discussing their decision to **adopt**. CAS was perceived to align with, and address, existing school priorities and/or concerns over pupils’ health ^(1.1)^, for example:

*“I know my head was very passionate about it because of the obesity levels obviously in Bradford and specifically the report coming out about life expectancy. I think that was the main driving force for us.”* P20, PE Lead

Similarly, many schools highlighted that CAS complimented existing school development plans and could help alleviate Bradford’s stark health inequalities by promoting more physical activity opportunities^(1.2)^. CAS was seen as **compatible**, meeting an identified need in school, and contributing to the school vision^(10.1)^. CAS Champions felt using local Bradford data on health, obesity and inactivity was a compelling approach to get schools on board:

“*I think health statistics are really good and obesity statistics, particularly in our district in Bradford, have been the most heroine to our schools….Because studies from America are great when they show the importance of physical activity. But like getting real-life Bradford data has been the most empowering so far*” P2, CAS Champion

School staff were aware of broader (community-based) projects in Bradford and felt CAS was complimentary, adding value as part of a systems-based approach “*bringing together the community better, hearing a bit more about the wider impact of how we’re making children more active”* (P24, Teacher)^(6.3, 10.2)^. Some schools admitted the financial incentive piqued their initial interest^(1.3)^. This was a driver for adoption within many schools, although not all, as some school stakeholders commented on the need for evidence-based decision-making to ensure that they were “*actually doing it the right way around*” (P32). Not all eligible schools adopted CAS, and Champions suggested that SLTs within the schools didn’t feel they needed additional support for physical activity provision^(1.4)^ and could not see the value of what CAS had to offer^(10.3)^. For example, P2 said schools not engaging typically felt they could “*do it themselves or they already feel that they’re active enough*” (CAS Champion).

Schools identified CAS as an **acceptable** programme, with anticipation of the benefits to the school and enthusiasm to get started ^(7.1)^. In-school CAS leads were eager to talk to their Champions to share their progress and plan future training and CPD opportunities. General **satisfaction** with the CAS programme was consistently mentioned by many^(13.1)^, for example: *“Creating Active Schools has been fantastic”* (P19, PE Lead). The formal CoP and encompassing networking opportunities provided by the CAS team were consistently praised^(13.3)^. Staff felt the formal CoPs provided opportunities to reflect on progress and connect with other schools. This resulted in the development of CoPs with reciprocal school visits and mutual support.

CAS Champions and strategic lead support were consistently praised by school staff^(13.2)^. This included providing support with CAS documents (e.g. the Planning for Change document), arranging visits to other schools to observe practices and changes in provision due to CAS, and providing CPD opportunities for staff to promote wider school buy-in^(7.2)^. The initial **complexity** of the programme and how it was operationalised was alleviated over time due to the support and flexibility of the Champions^(14.2, 14.3)^:

*“I just want to say that I wouldn’t have been able to do it without my CAS Champion with me. I’d still be just looking at this, so confused. He’s been really helpful.*” P21, Teacher

This tied into the adaptability of CAS to increase usability. School staff highlighted the flexibility of the CAS framework to find “*what works for your school*” (P37) and the support from the CAS team to simplify aspects of the CAS programme^(8.1, 8.2)^. P23 said, “*P1’s really good and he changed all the planning documents and made it very simple into a clear action plan that we chose*”.

School staff were aware that implementation of CAS required an iterative step-by-step approach to promote **sustainability** from the outset and embed CAS into the heart of the school. This meant that in-school CAS leads and other school staff were committed to taking the necessary time to make informed decisions about implementing changes and spending money^(5.1)^. For example, P20 said, *“it is a really slow process, it doesn’t need to be immediate, it is gradual, it’s sustainable”* (PE Lead). This awareness came hand-in-hand with the knowledge that CAS was facilitating a whole-school physical activity culture shift ^(12.1)^:

*“It’s a culture change, but it’s not a small culture change if you want to do this properly, it’s a massive culture change for the whole-school, for every member of staff that’s in there, for the children, for everyone, and it is a big deal and that’s a difficult thing to have to get everyone on board with immediately. I think because you’ve so many things given to teachers all the time that come and go that are not sustainable”* P39, PE Lead

### CAS implementation: challenges and solutions

There was limited visibility and **reach** of CAS in schools, identified by school staff, senior leaders, and CAS Champions^(3.1)^. P13 said: *“Well, I think my school… if you said CAS they might not know what you meant*” (PE Lead). To increase the reach of CAS, some in-school CAS leads reported that CAS-related training opportunities helped^(3.2)^. One school established a working group to help share responsibility and maintain momentum:

*“If you give it to a select group of people who are going to focus on it every year, all year….. next year, when those teachers move to a different year group, like our year six teachers go to year four, they can pass it on to the new teacher, and so on.“ P17*, PE Lead

For some participants, the existing school **culture** meant CAS was often perceived as synonymous with PE and sports ^(12.5)^. This was demonstrated by SLTs typically delegating the in-school CAS role to their PE lead^(12.4)^. Furthermore, there were misconceptions about physical activity and how it may replace PE time, rather than providing additional opportunities, for example, P1 said, “*we’re at risk of lowering physical activity behaviours in terms of accelerometer measurement because we’re going from 2 PE sessions to a PE session and active enrichment*” (CAS strategic lead).

Senior leaders and school staff shared concerns about their ability to prioritise CAS and demands of accountability associated with other curriculum areas as defined by Ofsted^(6.4)^. Schools focussing on core subjects believed they had less capacity to implement CAS compared to schools that “*have got their maths and English right… have a little bit more wiggle room*”(P3, CAS Champion). Similarly, in-school CAS leads highlighted **feasibility** concerns over implementing CAS due to their lack of time and competing demands^(9.1)^. For some, there was a sense of not taking full responsibility as they expressed wanting more contact from their Champions to prompt action.

The impact of COVID-19 presented both opportunities and challenges^(6.1)^ to implementation. Some staff members felt CAS-related activities “*did grind to a bit of a halt because of Covid*” (P20, PE lead). Conversely, CAS Champions observed improvements in engagement with some schools, for example: “*Because of COVID, we’re just in a better place and we’ve got better relationships, we know which schools are our best schools to work with and that want to work with us*” (P2, CAS Champion).

While SLT support for CAS was seen to leverage whole-school buy-in to the programme^(12.3)^, participants expressed difficulties with wider staff buy-in^(12.6)^. P7 explained SLTs have a significant influence on establishing school culture and supporting change: “*I think SLT are big drivers for any change in a primary school, I think they sometimes underestimate what a big impact they have across their school*” (P7, CAS Champion). Challenges included perceived capacity concerns, a lack of interest in physical activity and difficulties in making changes to their daily routine to incorporate CAS. For example:

*“I think they think, this is too overwhelming, you want me to go outdoors, you want me to stop what I’m supposed to be doing and do something completely different and be more active and how am I supposed to do that? I think it’s a lot of training and just educating them that this is a positive thing, it’s not adding to workload, it might make it a little bit easier.“* P22, PE Lead

Some school staff reported a mindset shift during implementation^(12.7)^. This included an increase in knowledge and understanding of physical activity and CAS. Staff’s perceived **self-efficacy** was improved, typically due to CAS-related training increasing their confidence^(15.1)^. For example, P13 said, “One *of the Year 3 teachers, she said, oh it was just brilliant, like the orienteering training and then to give her the confidence”* (PE Lead). There was an initial apprehension of CAS and the perceived programme complexity for both SLT and in-school CAS leads. Attending the first CoP was initially overwhelming due to the limited understanding of what CAS entailed and who else was involved, but over time this was alleviated^(14.2)^. The organised networking opportunities helped staff feel at ease when they realised other school staff were also getting to grips with what the programme entailed. In-school CAS leads that got involved later in the academic year did not share this confusion. Similarly, the administrative tasks (e.g. profiling tool) were seen as “*quite labour intensive let’s say particularly at the beginning, but it is useful*” (P29, SLT). However, with hindsight, staff benefitted from the clarity it provided to their current physical activity provision which allowed them to effectively action plan^(13.4)^.

At the locality level, there was variability among CAS Champions’ perceived **self-efficacy** about the role^(15.2)^. This was highlighted by the strategic lead due to their differing backgrounds and skill sets. Due to time pressures, there were limited opportunities for CAS Champion training before school implementation began^(6.6)^. The need for future training to better equip the Champions and provide parity in the type of support they could provide was acknowledged. This included additional training to support Champions in checking and challenging schools to ensure **fidelity** and integrity with the profiling tool and Planning for Change document^(4.1)^.

CAS Champions reflected that in-school CAS leads needed autonomy to make decisions, particularly to ensure that any changes made due to CAS would be **feasible**^(9.2)^. Where in-school CAS leads were unsupported or unable to galvanise commitment from wider school staff, CAS Champions highlighted that implementation was limited. Similarly, in-school CAS leads in some schools felt that without SLT support, their ability to create change was limited^(13.2)^. Finally, there were concerns over how staff turnover may negatively impact CAS implementation^(6.5)^. Drawing on past project experience, P35 said: “*I’ve been involved in a few [projects] over the years in Bradford and that happened, something comes out and it’s amazing and then the person that leads it goes and then it’s not… no one does it anymore.”* (SLT). Some staff had concerns about how to alleviate this as well as upskilling and training new staff each academic year. Similarly, SLT felt that **sustainability** was reliant on the continuation of CAS champion support ^(5.2)^. Conversely, a school that had amended its school development plan and school priorities were not as concerned with the CAS lead stating “*I know it’ll carry on at that school anyway so I don’t need to be there. I’m probably the man on the ground doing it, I know that [the head is] in the background doing it…”* (P40, Coach).

### Perceived effectiveness of CAS at the school level

The **perceived effectiveness** theme included positive changes across all four aspects of the CAS framework: school policy^(16.1)^, the school environment^(16.2)^, school-based stakeholders^(16.3)^, and increases in physical activity opportunities^(16.4)^. This included – in some schools – changes to school policies and development plans, as P18 highlighted “*So, we got written into school improvement plan that we would look at physical activity and the physical activity had to improve*” (PE Lead). Those on board with CAS as a sustainable approach to whole-school physical activity wanted to see more systemic changes taking place^(12.1, 16.1)^. That said, some staff members were disappointed to not see policy changes in their schools^(12.2)^. Influencing stakeholders and empowering school staff resulted in schools accessing additional funds which positively impact the environment:

*“By working with the staff as stakeholders, we’ve then been able to address the environment. So, for example, one of our schools has just a concrete playground. But because we worked with lunchtime staff there, we empowered them, we encouraged the play at lunchtimes, they’ve now got £50k worth of outdoor grants to build up their outdoor space”* P2, CAS Champion

There was a clear emphasis on increasing physical activity opportunities across most schools and Champions felt the same, often wanting to focus on policy but ending up discussing opportunities^(16.4)^. These ranged from “*active travel*” (P40, Health & Wellbeing Coach) to “*active enrichment*” (P23, SLT) and new afterschool clubs that were “*not football or cricket, it’s archery and nature and Jujitsu and fencing, sports they don’t normally do*” (P21, Teacher). School staff discussed the perceived impact on children’s physical activity levels and classroom behaviour^(16.5)^. One school used digital devices to track changes in steps, and another started to see behaviour benefits from incorporating movement breaks within the timetable to help “reset” the children.

## Discussion

This study used both the implementation evaluation roadmap [34] and CFIR [25] to provide insights into determinants that influenced the locality-based implementation of a whole-school programme focussed on creating organisational and cultural change for physical activity. CAS is the first whole-school physical activity approach that is informed by a co-developed framework [17], underpinned by both behavioural and implementation science. Findings demonstrated the willingness of schools to adopt and implement a whole-school approach when perceived as high quality and aligned with current school values. In contrast, schools identified challenges with wider school buy-in, shifting school culture and building staff competence. The CAS approach was perceived in a supportive light due to focusing on small incremental changes that were sustained over time, rather than short-term fads. Formal and informal CoPs provided “safe spaces” for support and developing new physical activity practices. The support structure of using CAS Champions and in-school CAS leads facilitated the change process across schools. Challenges remained with achieving wider reach within some schools, resulting in varied implementation. While some schools managed to facilitate policy-level changes, the majority of schools focussed on physical activity opportunities. To reflect the use of implementation science in the design of the CAS programme and promote transferability of the findings across whole-school physical activity research, the key findings are discussed in relation to the CFIR domains [25].

Aligned to the characteristics of individuals, participating school staff were enthusiastic to adopt CAS. Similar to previous research, wider staff buy-in was challenging due to a perceived lack of time and capacity [41, 42], rather than dissatisfaction with the programme. These findings suggest school stakeholders’ lacked readiness for change, which may be indicative of wider school culture. To broaden engagement, the diffusion of innovation curve suggests a need to demonstrate the value of CAS and increase programme support to engage early and late adopters [43]. That said, in-school CAS leads highlighted an improvement in their colleagues’ self-efficacy after attending CAS training. Positive changes in teacher and wider school staff behaviour to adopt and implement CAS were also observed within the same population in a complementary study focussed on assessing organisational change in physical activity [27]. Combined, there is a need for future whole-school physical activity programmes to broaden initial training beyond in-school leads. The challenge persists in how to deliver this at scale with a potential need for online programmes which have demonstrated effectiveness in previous school-based physical activity programmes [33].

Aligned with the inner setting, CAS was embraced by school SLTs and in-school leads after nine months. Similar to previous work, where SLTs were proactive in programme implementation, greater reach was achieved [44]. Given the focus on the initial nine months of the programme, we would expect broader reach to occur as time progresses due to the development of in-school CAS working groups, CAS training in school and increased opportunities for wider staff to attend CoPs. Essential to the implementation process was the assignment of the in-school CAS lead to a middle or senior leader. This was to prevent the conflation of physical activity and PE and to ensure higher-system support to facilitate the structural changes required as part of the CAS programme. Even so, 64% of the sample were PE leads, which may lead to the disengagement of wider-school staff due to CAS being seen as the “PE leads’ job”. Therefore, future programmes should develop strategies to overcome this issue if whole-school leadership for physical activity is to align with existing PE leadership. This issue links to a broader debate and the need to differentiate PE, school sports and physical activity at all levels of the school system [45].

Financial incentives encouraged SLT to adopt CAS, which aligns with CFIRs outer setting construct. Following initial uptake, SLT quickly understood the ethos of CAS focusing on sustainable changes towards whole school physical activity, which was demonstrated in the adoption reasons quickly moving away from the initial financial interest. Our findings align with a school-based physical activity initiative in Ireland where adoption was also incentivised through rewards [46]. While incentives positively influenced initial buy-in, a concurrent CAS evaluation identified no differences in programme effects between schools with and without financial incentives [27]. Concerning intervention characteristics, other adoption reasons aligned with schools perceiving CAS as a professional development programme that met their needs and priorities around enhancing children’s health outcomes. Specifically, the use of local obesity and premature mortality data enhanced CAS adoption. While data with statistical power and larger sample sizes offers rigour within the research world, producing and/or identifying local data may be of greater benefit to increase adoption rates and in-school buy-in. Linked to the intervention characteristics (e.g. relative advantage, cost), for some, adoption was initially fuelled by incentives, yet as time progressed, recognition that CAS could genuinely create systemic change for physical activity influenced sustained implementation.

Similar to previous work, the CAS implementation processes were valued by schools as they were able to trial strategies and take a step-change approach [47], making small sustainable gains each academic year. This was supported by schools showing an appetite to commit to CAS long-term, given their appreciation of CAS as a system change approach, demonstrating a positive culture within the inner setting. Similarly, the SWITCH wellness intervention - underpinned by implementation science - reported a shift in school culture, with schools sharing their plans to continue the programme despite experiencing challenges [32]. Combined, these findings support a need to move beyond single and multi-component approaches [22] to programmes that address higher system factors (e.g. such as policy and environments) that are characteristic of whole-school approaches.

The formal networking opportunities - as part of the characteristics of the intervention - were essential for providing programme and peer-to-peer support and reducing perceived programme complexity. Within and between schools informal CoPs emerged to support staff to implement physical activity opportunities and implement higher-level systems change. As seen in previous research, this support offers an effective and sustainable way to continuously learn from one another [48]. CAS Champions provided the greatest influence at the school level through their relationships with in-school CAS leads and by brokering school-to-school opportunities to share learning. Similarly, previous research has highlighted the need for practitioners to engage in a CoP [49–51] to help share passion and enthusiasm for the whole-school approach with peers in a supportive environment [52].

### Future recommendations

Analysis using the CFIR [30] and the implementation evaluation roadmap [34] provides an integrated understanding of implementation determinants across the different school-based domains; providing valuable insights for future research and practice. While incentivisation was important to some schools for initial buy-in, the perception of the programme as a professional and comprehensive approach to creating effective systemic change, promoted ongoing engagement. Future research is needed to better understand the implementation determinants of whole-school physical activity approaches across wider contexts, both within and beyond the UK, to gain broader insights. Further, essential work is required on how to reduce the initial intervention burden and perceived complexity of whole-school approaches, which, in turn, may increase the reach within schools. This can be achieved by understanding how we can support school stakeholders who have initial apathy or resistance to physical activity.

### Strengths and weaknesses

The current study is the first of its kind to use the implementation evaluation roadmap [34] framework to understand implementation determinants within a whole-school physical activity programme. Moreover the use of CFIR (as a determinant framework) and the implementation evaluation roadmap (as an implementation evaluation framework) provided a comprehensive approach to examining implementation concepts and constructs. Such an approach has been advocated when conducting population health intervention research [53]. Some limitations should also be noted. While CAS is designed as a multi-year programme, the implementation evaluation has taken place for the initial nine months where adoption and sustainability considerations were high. Additional investigation is warranted to track changes to implementation outcomes and determinants over the intended scope of the program. As data collection took place at a formal CoP, the sample was limited to schools that may have been engaged in CAS at a deeper level. Our sample did not include schools that decided not to adopt. Despite this, CAS Champions were involved in the initial recruitment and were, therefore, able to report on schools that did not adopt CAS. Furthermore, while we spoke to one-to-two representatives per school, which has produced insights at the whole-school level, speaking to more in-school staff may garner greater insights at the stakeholder level. Finally, the current project offers contextual findings based on the implementation of CAS in the Bradford locality. Bradford was chosen as it is one of the more diverse and poorly resourced areas of the UK, which provides a challenging environment for adoption. Despite this advantage, Bradford also represents a narrow setting and investigating additional localities will support wider dissemination across differing contexts.

## Conclusion

The present study reveals novel insights on the implementation of CAS, specifically aligned to adoption, reach and sustainability. The success of CAS implementation in promoting a positive cultural change for physical activity was determined by programme acceptability, intervention complexity, school culture and school stakeholders’ perceived self-efficacy. Drawing on the implementation evaluation roadmap [34] and CFIR [25] established a rigorous method to evaluate a programme underpinned by behavioural and implementation science; progressing the current understanding of whole-school approaches to physical activity. Replicating these methods in similar whole-school approaches to physical activity would improve comparisons in the field and help bridge the gap in understanding the voltage drop in effectiveness when scaling up interventions from efficacy.

## Electronic supplementary material

Below is the link to the electronic supplementary material.

Supplementary Material 1: Interview Guides

Supplementary Material 2: Comprehensive CAS Themes and Sub-themes Aligned to the Implementation Roadmap and CFIR

Supplementary Material 3: Standards for Reporting Qualitative Research (SRQR) Checklist

## Data Availability

Summarised data generated and analysed during this study are included in this published article and its supplementary information files.

## List of abbreviations

CAS: Creating Active Schools
CFIR: Consolidated Framework for Implementation Research
CoP: Communities of practice
