# Supplementary material for: Initial insights into the impact and implementation of Creating Active Schools in Bradford, UK
Source: Int J Behav Nutr Phys Act. 2023 Jul 5;20:80. doi: 10.1186/s12966-023-01485-3 (PMC10320983; doi:10.1186/s12966-023-01485-3)
Supplement: Supplementary file 1 — Supplementary Material 1: Interview Guides [file 12966_2023_1485_MOESM1_ESM.pdf]

INTERVIEW GUIDE FOR CAS CHAMPIONS/FACILITATOR

Ensure copies of the framework are available in the focus group so that you can use this to facilitate the questions; specifically using prompts on policy, environments (social & physical), stakeholders and opportunities.

Ice-breaker questions

1. Can you tell me a little bit about your current job role and experience with the Creative Active Schools (CAS) Framework?

Adoption- describe as signed letter of agreement, CAS in-school lead allocated and engaged staff in whole-school session.

2. What do you think have been the key drivers/motivation for schools who adopt CAS?
3. Can you tell me about any changes, if any, in the schools' key drivers/motivations throughout the first year of the programme?
    - Prompt before CAS started, in the first 6 weeks and now.

Effectiveness

4. Can you tell me about any impacts CAS may have had on the provision of physical activity, PE, and school sport within the schools you are working with as a CAS champion? *(have CAS framework on show)*
    - Prompt: Policy, Environments, Stakeholders, Opportunities
5. Have there been any impacts of CAS beyond the individual schools?

Implementation

6. Thinking about your answer on impacts in school, can you tell me **WHAT** you think has supported schools to change their provision?
    - Prompt: Think about the different elements of the CAS offer (profile tool, CAS champions, communities of practice etc).
    - Prompt: Is there anything that is not "officially" part of CAS that has influenced the changes?
    - Prompt: What about In-school and out of school support?
7. Referring to your previous answer, **HOW** have these factors supported schools?
8. Can you share any examples where you think CAS has not worked so well? What may have been the reasons for this?
    - Prompt: Policy, Social and Physical Environments, Stakeholders, Opportunities
9. Thinking about the staff who have changed provision within their schools. What are the skills/characteristics that have helped facilitate this change?

School resistance characteristics

10. If applicable, can you tell me about any similarities/key characteristics for schools that have either not adopted CAS or quickly dropped CAS following adoption?

CAS champion (if you have time, ask these questions)

11. [If not captured in earlier questions…] Reflecting on your role as a CAS Champion, can you tell me what you feel has enabled schools to better succeed with using the CAS framework?
12. What skills do you think you require to be an effective CAS champion?

INTERVIEW GUIDE FOR CAS LEADS, TEACHERS AND SLT

Ensure copies of the framework are available in the focus group so that you can use this to facilitate the questions; specifically using prompts on policy, environments (social & physical), stakeholders and opportunities.

Ice-breaker questions

1. Can you tell me a little bit about your current role and experience with the Creative Active Schools (CAS) Framework?

Adoption: describe as signed letter of agreement, CAS in-school lead allocated and engaged staff in whole-school session

2. Can you tell me why your school signed up to the CAS framework?
3. Can you talk to me about your journey with signing up to CAS?
4. What has the response of other teachers in your school been like?
    - Prompt: Any distinct positive/negative reactions to the use of CAS?
    - Prompt: Have there been any changes in teachers' reactions over time?

Effectiveness

5. Can you tell me about any impacts CAS may have had on the provision of physical activity, PE, and School Sport within your school?
    - Policy,
    - Environments,
    - Stakeholders
    - Opportunities

Implementation

6. Thinking about your answer on the impact of CAS in your school, can you tell me **WHAT** you think has supported you to change your provision?
    - Prompt: Think about the different elements of the CAS offer (profile tool, CAS champions, communities of practice etc)
    - Prompt: Is there anything that is not "officially" part of CAS that has influenced the changes?
    - Prompt: In-school and out of school support.
7. Referring your previous answer, **HOW** have these factors supported your school?
8. Can you share any examples of challenges using the CAS approach? What may have been the reasons for this?
    - Prompts: Policy, Social and Physical Environments, Stakeholders, Opportunities
9. Can you talk about the role the CAS champions and CAS programme manager have played in supporting your school?
    - Prompt- what have they done that has helped use CAS?
    - Prompt- Are there things they have not done that you would like them to do?

End

10. Is there anything else that we have not asked that you would like to add?
