# Supplementary material for: Initial insights into the impact and implementation of Creating Active Schools in Bradford, UK
Source: Int J Behav Nutr Phys Act. 2023 Jul 5;20:80. doi: 10.1186/s12966-023-01485-3 (PMC10320983; doi:10.1186/s12966-023-01485-3)
Supplement: Supplementary file 2 — Supplementary Material 2: Comprehensive CAS Themes and Sub-themes Aligned to the Implementation Roadmap and CFIR [file 12966_2023_1485_MOESM2_ESM.docx]

| **Construct** | **Theme** | **Quotes** | **CFIR alignment** |
| --- | --- | --- | --- |
| Adoption | Attractiveness of the CAS Ethos | “But one of the main things I think was see the obese levels in the school. You see the kids that are not taking part in physical activity, you'd like them to get and trying to get a way around that in an area that is very, very deprived, unfortunately. The more I looked at it as well, I thought for me who is sporty and I've always loved sports. Understanding that not everyone does like sports, how do you get those people to realise the importance of sports? When there not that bothered about it so it’s getting ideas on that and to share practice one that, that’s really useful." P27  “I think the schools that have engaged is probably been the data I guess from Born in Bradford because the statistics on sedentary behaviours, lack of activity, are quite damaging I think, that sort of captures people’s attention especially when they’re going into schools and you’re a little bit nervy in terms of how they’re going to respond as a staff team, but I think you can’t really ignore those numbers, so I think yeah, definitely the data on levels on inactivity across Bradford.” P6  "I know my head was very passionate about it because of the obesity levels obviously in Bradford and specifically the report coming out about life expectancy. I think that was the main driving force for us." P20  “One thing that’s really worked for us and the message I’ve got across is that 10-year gap, that’s resonated with everyone, the Living Well video that talks about the 10-year gap between Waterdale to inner city Bradford, it’s unnerving people and they just go, ‘That’s wrong!, I work in Bradford and I see those children’.” P7  “We were determined because it was already on our school improvement plot that we like this idea of the whole child for the whole day. And we realised that the children were not active enough.” P26 | Outer setting   - Patient needs and resources |
|  | Existing Commitment to a PA Agenda Though Locality-based Projects | “All of our schools had to come on board, because our offer for physical activity is the RIC programme. So, when they were invited to the programme, the offer was CAS and everybody was on board from the beginning” P2  “…we've been in commissioned neighbourhoods where the JU:MP part of the project has been run by the council.” P28/9  “we were taking on 30 new schools through the accelerator programme in JU:MP, but also these Living Well, reducing inequality in the city (RIC) schools. And there's an overlap between those as well.” P1 | Outer setting   - Cosmopolitanism - Peer pressure |
|  | Initial Financial Incentive | “Do you want me to be completely honest?.... Because you were going to give me £10,000 to put something nice in my playground and that’s a big pull and that was the hook in, and then you take it from there, don’t you?” P32  “The bottom line is the first thing that encourages people along is the opportunity for funding…” P6  “What’s really funny is, I thought that I would spend the ten grand and then fizzle out with the programme, but actually, we’re taking our time with the programme, and we haven’t even got to the point of the money. So, we are actually doing it the right way around…. So, what initially was very money-driven has actually taken a turn, not because anybody’s decided to do that, but because of what I said earlier about the clunkiness in logistics and communicating.” P32 | Outer setting   - External policies and incentives |
|  | School Implementation Readiness | “…When I first started. I didn’t realise how big it was going to be. I did have a phone call with P1 before signing up, and he went through all what it was going to look like, but I think it took me a few JU:MP meetings to fully comprehend just how big and wide-scale the whole project is.” P34  “I don’t know if other members of staff quite understand how big, I think especially as you say when we signed on, it was basically email said £10,000, come to this meeting. Oh great, and then Head Teacher was straight away… and the Head Teacher now I think, understands more of how good, but if you look at other members of staff, they probably don’t realise how big and broad a scheme it is, and exactly what’s going on.” P14 | Intervention characteristics   - Complexity - Cost   Process characteristics   - Executing |
| Dose delivered | CAS Champions Facilitating Delivery | Successes  “We did some training to say we were going to get the children to be more active. Then P28/P9 and her piece came and did some training and the staff were on board really quickly” P26  “Then the main process for CAS was support from the CAS champions both in completing that online tool, then coming up with their plan. And then looking at what were their implementation strategies and what support they need to actually make them happen. And this is where we got variable quality because of a few things” P1  Areas to improve one  ‘I would probably benefit O kind of contacting us a little bit more and just kind of saying ‘oh we’ve not met up this half term, can we get a date in the diary?’ P37  "I’ve never had a meeting with P6." P19  "So, have you been told that P6’s your CAS champion or not?"(P18), "Not a clue." (P16)  "I haven’t had a meeting with P6 or anything like that." P19 | Process   - Champions - Engaging |
| Reach | Limited Visibility & Permeation of CAS in Schools | "Well, I think my school, they probably, if you said CAS they might actually not know what you meant.’ P13  “I feel like there isn’t still enough of a profile with JU:MP in our school. I think, if you came in tomorrow and asked our children, what is JU:MP, the vast majority still wouldn’t know.” P33  “I suppose as well, it’s a little bit like, you know, when someone comes round for a tour, and it’s like, who’s that, who’s that? It’s like, you need to be like, well, that’s the CAS team, to implement the change. They’re not involved directly, but they know that something’s happening.” P31  “It’s kind of happening in my classroom and not really getting much further and obviously we share planning on our corridor so I’m planning active things into those lessons, so that’s another two classes, but after that lesson they’re not then planning like that in their other lessons, so I just feel like we need more bodies on it I think.” P37 | Inner setting   - Networks and communications - Implementation climate   Process   - Executing |
|  | Identified Approaches to Increase Reach of CAS | For SLT:  “I think when you hear from current head teachers, especially the ones that have been massively influenced in terms of their mindset shifted completely, I know this is a small sample, I think about my head teacher and the chap who was at the last conference…. His kind of 10/15 minutes, if you had head teachers in the room and he talked so openly and honestly in front of like a bunch of head teachers, I think we’d see a much wider impact.” P6  For Staff:  "One of the Year 3 teachers, she said, oh it was just brilliant, like the training and then to give her the confidence to do it….” P13  "Ours, we’ve set up a working group for staff. Because I used to push the activities and things as a PE leader. It was just too much for one person to do… So, one person would teach in every year group who knows what the CAS framework is and what it represents. We’d far rather do it to every single member of staff, but it just, from experience, it just doesn’t work. If you give it to everybody, it just doesn’t work. If you give it to a select group of people who are going to focus on it every year, all year….. next year, when those teachers move to a different year group, like our year six teachers go to year four, they can pass it on to the new teacher, and so on and so forth." P17 | Inner setting   - Leadership engagement - Networks and communications   Process   - Engaging   - Opinion leaders |
| Fidelity | CAS Champions to act as Friendly Critics to Schools | Champions need to critically challenge schools to ensure profiling tool is completed with integrity:  “We said, you've [the champions have] got to help the schools complete the profile assets, then we then did a session on how they constructively challenge schools on both their answers in their planning. We then then remit again, and all of the CAS champions, effectively with the exception of one and just said, Oh, I just accepted what the school have done. And so we had to sort of rewind a bit and go, but actually, they're not using the framework. They're not using the profile at all, they've almost just gone, we want to do this. And we need to be not saying you can't do that, but giving them you know, we've given them the question toolkit to ask those questions, and they're not really done it” P1  “[The profile tool] provides a little bit of accountability, I think as well then rather than thinking, oh, we started that but we're too busy with phonics and reading it. If you know that some of them come back in to see wherever you got to you are probably more likely to do something and act on what you spoke about previously. So, it's always useful to us.” P27  Need the CPD tools to make schools more accountable:  “I think as well I know we couldn’t start with the CPD modules because they’d not been developed but it’s almost like a physical hook, whether that be going in to see a school in action that’s already established some sort of physical activity or those CPD modules almost compare initially as a menu so schools can obviously tap into what they think they need support in, obviously there’s a lot of talk about toolkits and we’re still kind of talking about them now. I think that needed to be done as early as possible to keep the schools engaged, particular school leaders, they want to see something happening, not as P4’s referencing, lots and lots of emails going back and forth, there needs to be something practical starting to take shape.” P6 | Intervention characteristics   - Design quality and packaging |
| Sustainability | Implementation Efforts to Ensure Sustainability | General sustainable comments:  “If we're going to do it, we might as well take it on properly and try have some sort of continuity plan going forward" P27  “It’s clearly a long-term project, there’s a lot that can go into this, so we need to understand that you need to decide, we’ve got focus areas for this year, so plan for next year and understand that it’s a bit of a longer project but the end goal and the end product should, yeah, really be beneficial for everyone particularly.” P39  Stakeholder-based sustainable changes (development of a community of practice within the school):  “So, like, next year, when those teachers move to a different year group, like our year six teachers go to year four, they can pass it on to the new teacher, and so on and so forth.” P17  Opportunity-based sustainable changes:  “So things are becoming more of a habit that will just hopefully keep ticking on like that. And again, like with the scooters, we’ve yet to do… we’re going to try and do a scooter club, but once you do one, once you’ve done it once, then you can keep… so just all these things right, so it’s a bit of a tick list, but once they get going in the school, I think that’s when you’ll see the impact.” P13  “One of the best pieces of advice that I think P1 gave was, it is a really slow process, it doesn’t need to be immediate, it is gradual, it’s something that’s sustainable. We’ve had B in as well, we’ve focused on the active elements of lessons. We’ve had a JUMP Day but that was just before Covid hit. Now, we’re starting to try and get things back on track." P20 | Process characteristics   - Executing   Intervention characteristics   - Adaptability   Process   - Planning |
|  | CAS Champion Support a Necessity for Sustainability | “That’s my concern about it going forward when it just becomes a support system, a base, without money attached to it, will the level of rigour and interest in it remain the same.” P5  “Even if it’s not going right the CAS champion support is still there to pick it back up and take it in a different direction. I think even without the funding, if you’ve got a CAS champion there, that’ll be the most beneficial link.” P4 | Process characteristics   - Champions |
| Context | Opportunities and Challenges Provided by Covid-19 | Momentum for CAS related work lost:  “Yes, it did grind to a bit of a halt because of Covid… We’ve had a JUMP Day but that was just before Covid hit. Now, we’re starting to try and get things back on track." P20  "I think we had because we launched it and it was January time when we were just having COVID and we had to do the staff meetings over Zoom and it didn't really have the impact that we wanted. So, I think it's sort of fallen a bit flat because of that and you don’t hear, there's not really a pause or anything with staff. So, I think what we need to do is sort of launch that and try to infuse staff a bit more because at the moment, they're not on board. Yeah." P30  “We’ve also been hit with COVID as a school twice this year, even though apparently the pandemic’s finished, which has seen my PE lead off twice and her support staff off. We’ve then found that the dates and trying to make the arrangements for the launch day with parents, that’s been quite troublesome.”P32  Opportunities:  “Because of COVID, we’re just in a better place and we’ve got better relationships, we know which schools are our best schools to work with and that want to work with us. So, we’re just in a good place to really embed more now, I think. And we’ve learnt a lot too, haven’t we? About what’s worked” P2  “It’s helped us with so many things. You wouldn’t… You know, COVID, for our school, as much as we all, we killed off a community at Christmas, which was interesting, there were so many positives came from COVID from infecting teachers who then went on to go elsewhere, um, to the uniform, the PE uniform, to parental engagement. I think also, parents…” P32 | Inner setting   - Implementation climate |
|  | Impact of Financial Differences Between Schools | CAS Champion awareness of schools with and without funding and the resentment that might be forming:  “Maybe that’s the thing with our schools is that a lot of half schools are getting funding from CAS to develop it. But then a lot of our schools have zero pounds funding, so we are the funded element of it, the facilitation offer, so everything has to be free. If they want to send staff out, it’s at their expense, which is why it’s difficult in the district, because the funding can only go to some schools and not to others. And then we found that when we’re in big CAS conference events and they’re hearing about all this funding and all this opportunity because of money, they don’t get any of it. It can be a bit disheartening for them and whilst we try and give them as much of our time to prove that you do have us and we can come in and do all this for you. But it’s not the same when they’re getting fancy bits of equipment and stuff and they’ve already stretched their PESPA budgets each year, because there’s no funding. And there’s you know talk of £50,000 grants flying round it’s… I could imagine, you know, it’s a bit disheartening and even like on Twitter they all see other schools boasting about what they’ve got and what they’ve done and opportunities they’ve been able to give and fancy forest schools. And then ours don’t get any of that.” P2  Awaiting funds  “I think without funding though, because the funding’s waiting now we haven’t got that money in the bank, then you can’t start these events in September, it has a knock-on effect.” P15  “What I found was harder, some schools have had funding, and some schools haven’t.” P12  Perception of needing funds to act on CAS:  "Yeah, so just having specific opportunities to do things really, and then like the fun days again, like you mentioned, it’s just like we need to have the funding. Because that was one specific wasn’t it, you need to have the funding, this needs to happen, what we do for funding." P13 | Outer setting   - External policy and incentives - Cosmopolitanism |
|  | Added Value of CAS to Other Locality Initiatives Addressing Health Inequalities in Bradford  -  Processes & Outcomes | "I think the neighbourhood forums is a good way of networking, if you know who to get in contact with Emily who has been able to rollout our CPD from scooter training, balance bike training, so that plays into it as well. " P14  “And it’s actually quite nice to see that there are different stakeholders present and they’re actually making a difference for the children because they’ve got the same vision and they’ve been very proactive and optimistic about it and it’s nice to see that. I don’t think that sometimes, as teachers in a school, you get to see the bigger picture. Now we’re so involved in it.” P23  “It’s that kind of bringing together of the community better, hearing a bit more about the wider impact of how we're making children more active.” P24  “we’re quite an isolated school in terms of we don’t really have any partnerships with any other schools or really within the community. So the neighbourhood meetings for us have been really great and our headteacher’s actually attended the majority of the neighbourhood meetings, and I think that’s been something that has been really important, and I think that’s a wider reach for us, wider than the physical activity.” P9  “I'm not as convinced yet about the JU:MP stuff as I am with the CAS stuff.” P26  “I mean, my view, talking as my personal view here, is that CAS in unbelievably important, and I’m struggling to see the benefit of JU:MP. CAS as a tool for school improvement, phenomenal.” P33  “There was a school with one of the best physical activity leads I’ve ever come across in my teaching career, and they gave in pretty early, and my interpretation was due to the JU:MP meetings, and it being just not purposeful, wasting a very skilled gentleman’s time, because I think he was sat there like ‘I don’t need this’ whereas actually, possibly if we’d have kept him he might’ve been a bit like myself where actually he might’ve developed even further because of it, but actually their school was physically active anyway; we don’t need to be continually coming to these meetings where we introduce ourselves before the meeting starts.” P5  Outcome: “One of the greatest things about JU:MP, the real power of it is this principle of getting the communities to work with the schools, that CAS and JU:MP link. That is the winning combination for health in Bradford is getting schools to work with the communities and actually know what’s going on” P7 | Outer setting   - Patient needs and resources   Intervention characteristics   - Intervention source - *external* |
|  | Ofsted Priorities Misalign with CAS | “I think the obstacles, as with everything in school, is the fact that they have to pass the SATs, and that’s ultimately what Ofsted are looking at isn’t it, for data for all schools. So if CAS was as important as that” P13  “it’s a good school and they’re a really well-established good school, whereas [X CAS school] has started a project, we’re waiting for Ofsted, the priorities of the two is very, very different. So I feel like [X CAS school] have got their maths and English right and that’s one thing, so they’ve got a little bit more wiggle room, a little bit more space to think ahead about things wider, whereas we were in a position where actually we had to really focus on those maths and English and these core subjects.” P3  “It is such a shame actually, so Ofsted did come this year and we moved from RI to good and they did a deep dive in PE, I feel like the work that we’ve done with CAS and stuff really helped towards that and it would just be so nice if there wasn’t that pressure towards the maths and English and getting all that completely right, because then it could be used as like a tool… physical activity is a tool for school improvement, that would be really nice, but I don’t think… I think it’s a very long way off.” P9  “I have spoken to three or four head teachers, were there just said, I've changed my mind about wanting to do this, and I definitely want to do it, but we're on our knees. And I can't do it now. And I can't I don't actually foresee being able to do it in another year or two, because I'll start this, you know, and so all of those other factors like staffing budget, pressure from Ofsted, and even though they've been persuaded that this is an agenda to focus on” P1 | Outer setting   - External policy and incentives |
|  | Staff Turnover Implications on CAS Implementation | “I know it’ll carry on at that school anyway so I don’t need to be there. I’m probably the man on the ground doing it, I know that she’s [the head] in the background doing it…” P40  “I’ve been involved in a few [projects] over the years in Bradford and that happened, something comes out and it’s amazing and then the person that leads it goes and then it’s not… no one does it anymore.” P35  “We’ve already seen the dynamic change a little bit with our relationship with them, because she’s not there anymore. And it’s not that they’re not involved, they were at the CAS conference and everything. It’s just different because we don’t have such an in road into the school anymore. I think as staff move and some of our RIC leads, CAS leads in the schools are moving. So, it’s like what happens now? How will our relationship look next year?” P2 | Inner setting:   - Culture |
|  | Limited Opportunity for CAS Champion Training | “I think if we have more time, there's a lot we could do with the CAS champions to develop them professionally so they're in a better place to deliver on what they need to do… . But again, not that I'm throwing out all the challenges, but at the reality is that the capacity we've had, with a very small team that working with that many schools is we probably needed better, better training for the CAS champions and more of it before they then went and did their role. Whereas they effectively had two days and then they were running this process.” P1  “Yes, and we’ve been having our CAS champion training, tends to be the last week or two of a half term or term, for what we’re going to… For the support we’re then going to give the following term to our schools. So, it has been very much short term.” P33  “The reality is that the capacity we've had, with a very small team that working with that many schools is we probably needed better, better training for the CAS champions and more of it before they then went and did their role. Whereas they effectively had two days and then they were running this process” P1 | Process characteristics   - Champions   Intervention characteristics   - Design quality and packaging |
| Acceptability | Initial Enthusiasm and Anticipation for CAS | “They’re really excited and enthusiastic. They talked about loads they wanted to sort out in September with O and they were like chomping at the bit to come and speak to me and tell me about what they’d done, and like the teachers, they could all kind of understand why they’d want to put this into practice and why we’d want an active school. Like I say, it’s just implementing that now and pushing it forward.” P36  “I think being part of CAS has been really helpful for our school as well, because we certainly are at the very beginning of making our school more active, at the beginning of that journey, so it’s been really useful for me to be able to come and network with all these different people, but also learn so much” P9 | Characteristics of individuals   - Knowledge and beliefs about the intervention   Inner setting   - Networks and communication |
|  | CAS Champion Support Promotes Wider School Buy-in | “The funding is great in the first instance but having the support of the CAS Champions, I think that’s more crucial. I think, as I say, I’ve been in fairly regular contact with some of my schools, even schools that are out of my neighbourhood that I’m supposed to be working in, but I think it goes back that initial point of these schools need to see something that’s happening that works, and that can almost inspire them to invest the funding because as much as we sit around the table and talk about what that funding can be invested in, unless you actually see it happening successfully somewhere, it’s quite difficult to justify it.” P6 | Process characteristics   - Champions |
| Adaptability | Simplification of CAS tools to Increase Useability | “It’s not a set framework, we’re all in different positions, and every school is different.” P13  “We’ve simplified it [the profiling action plan] and it’s a little bit easier now for us to implement” P24  “So, P1’s really good and he changed all the planning documents and made it very simple into a clear action plan that we chose” P23 | Intervention characteristics   - Trialability - Adaptability - Complexity - Design quality and packaging |
|  | Flexibility in the School Delivery Model | “Staff were on board quite quickly and we tried a lot of stuff. And what we thought was rather try and plan it and get all right, we tried a lot of stuff and see what work… some of the stuff we've made mistakes, we've not got right and we've stopped doing and then some other stuff that we want to try in the future.” P26  “A bank of resources that they can tap into rather than saying they have to do this, so what works for your school” P37  “I mean we’ve looked at it, we will implement what we need, what we think we can sustain, we’re not into one trick pony stuff, so we’re doing what we think, we’ve really jiggled around, with the PA lessons for example haven’t we, and that suits us, and it’s how we can manage it.” P35 | Intervention characteristics   - Trialability - Adaptability   Inner setting   - Implementation climate |
| Feasibility | Staff’s (limited) Capacity | "I was going to say workload, it’s been actually I feel like probably again, because we didn’t do much for years, so maybe it’s not, but there’s lots to do and organise, that I’ve, compared to last year, I’m doing so much more.” P13  “Time is the crux of it because luckily I can afford to do that like you in a similar way but as a class teacher we appreciate that there’s not enough time at all” P38  “Then there’s another school that came to the events, they found it really beneficial, they want us to still help them with the schools and implementing physical activity, but they don’t have the time or the capacity to send the staff to the training or some of the events. Even though they would love to, which is what they’ve said to me, but they just don’t have the time.” P3 | Inner setting   - Readiness for implementation - Available resources |
|  | CAS Leads Autonomy to Make Decisions | For me, just the person who is the CAS lead needs to have the ability to make decisions. It’s as simple as that. So it doesn’t matter whether it’s the sports coach, a class teacher, the assistant head, the deputy head, as long as they are allowed to make decisions, like the ethos where I work is very much if you are the leader of that subject, you, you are making the decisions, whereas at a lot of schools it seems to be where you’ve got the two extremes, where it’s either the ethos of our school or the ethos of the school where you are micromanaged and every move you make is fine tooth-combed. And actually that’s where my frustration has been. Some of the CAS leads who were the most capable who I’m working with, are the people who’ve actually made the least progress, not through their fault, through people above not allowing them. So that’s so frustrating.” P6 | Inner setting   - Leadership engagement - Culture |
| Compatibility (appropriateness) | CAS Meeting an Identified Need in School | “I’d really like to see a policy from a school that’s got a policy on proactivity, because I can pretty much know that all of our schools sitting around this table, they do physical activity, but they’re not actually policy on physical activity, where does it fit within this, where is the training, they know about physical active lessons, and a creative curriculum balance, and that’s probably where we get in a lot of physical activity input I suppose. I’d like to hear somebody stand up there and say, this is our policy that we’ve shown the governors and it’s been approved.” P12  “I think policy for me is the big one. Because it’s always the hardest to address and even if we go in there with the aim of addressing policy, we end up addressing an opportunity.” P2  “It’s in our school development plan, one of the priorities was physical and mental wellbeing. The opportunity to be involved in this was exactly what we needed." P23  “if I look at some schools, I think it's the senior leadership team have prioritised well-being and therefore they see this, this dovetails nicely into that” P1  “We were determined because it was already on our school improvement plan that we like this idea of the whole child for the whole day. And we realised that the children were not active enough. So, really, it's been fairly easy really” P26 | Inner setting   - Tension for change - Relative priority |
|  | School’s see Value of CAS  *-*  *To alleviate health inequalities, promote PA opportunities, implement a whole system approach* | “One of the main things I think was see the obese levels in the school. You see the kids that are not taking part in physical activity, you'd like them to get and trying to get a way around that in an area that is very, very deprived, unfortunately.” P27  “So, our rationale for joining up was that we serve a community where we feel that we could promote greater physical activity. We feel as though there are more opportunities for our children, both within school and outside of school and we hoped that CAS was a way for us to make that a reality to execute them.” P29  “I think the whole systems approach is very attractive to schools as well, because they always talk about, oh, we have this intervention that comes in one year and then they try something new the next year and there’s always new things that come in. But this kind of encompasses them all together and puts them in one approach together which is attractive to schools, I think.” P2  “I think health statistics are really good and obesity statistics, particularly in our district in Bradford, have been the most heroine to our schools….Because studies from America are great when they show the importance of physical activity. But like getting real life Bradford data has been the most empowering so far, I would say” P2 | Intervention characteristics   - Evidence strength and quality |
|  | Incompatibility and Less Perceived Value of CAS | “So, often if they’re not engaged in CAS, that’s why, because they’re doing other stuff, but it’s just not CAS.” P2  “They were definitely in a position where they were a very sporty school and they’ve got PE and sport nailed down and that was sorted, they kind of were under the impression that they were a very physically active school.” P9  “For me there’s a couple; one in particular have got a real kind of sporting pedigree and I think the lack of understanding around the project to get it goes back to they’ve seen the money, something to do with activity, that must mean the school’s sport, let’s sign up, and again probably intimidated by the level of expectation and actually it’s so far from sport.” P6  “So, the ones that haven’t engaged, more recently, they’ve come to the first few CAS events and then they’ve decided that they can do it themselves or they already feel that they’re active enough.” P2 | Inner setting   - Tension for change - Compatibility |
| Cost | Financial and Opportunity Costs of releasing CAS Champion | “For the next academic year my head has said he won’t allow for release time, and he won’t pay the overtime anymore because he doesn’t see the benefit in what he’s gaining from it.” P10  “One of the things they flagged with me a couple of times was we need we want to do team, we want to like go and deliver this together. And I'm like, that's fine. Can you both get release time at the same time, are your schools because, again, we can't fund and there's limited funding for their time. And if we start funding them to do joint things, then it makes it more difficult” P1  “So, it’s easy for our schools to free us up, isn’t it? My school can release me for today, and it benefits my school and it benefits CAS, because I’m here as a CAS champion....And my school get the £250 or whatever it is for my time for today, but your school are paying your wages today, and your school are paying your wages today.” P18  “And adding to that as well, so this here, I’ve been the JU:MP lead for my school, I’ve done CAS and I’m also the maths lead at school and I’m a class teacher, so it gets to a point where you’re just like, I actually don’t want to leave more supplies for my class, because I’ve not been in my class three times this week, and it does… that’s on me taking that responsibility on, but maybe at the beginning I didn’t have that foresight, you know you can look back on things, and actually that’s fine, I can pay, I’ve got the money, I can pay for this and not be in class, but what does that have on my workload as a teacher, especially if you’re just a PE lead in school, it might be fine to take a bit of time out here and there, but sometimes to really have the impact and really do the work, it does take a lot of time.” P9 | Intervention characteristic   - Cost   Inner setting   - Available resources |
| Culture | Recognition for Whole School Culture Around Physical Activity | “it’s a culture change, but it’s not a small culture change if you want to do this properly, it’s a massive culture change for the whole school, for every member of staff that’s in there, for the children, for everyone, and it is a big deal and that’s a difficult thing to have to get everyone on board with immediately. I think because you’ve so many things given to teachers all the time that come and go that are not sustainable” P39 | Inner setting   - Culture |
|  | Idealism of School PA Policy (Currently Missing in Schools) | "Well lots has been provided by CAS, but the policy part I’m looking back here again, I’d really like to see a policy from a school that’s got a policy on proactivity, because I can pretty much know that all of our schools sitting around this table, they do physical activity, but they’re not actually policy on physical activity, where does it fit within this, where is the training, they know about physical active lessons, and a creative curriculum balance, and that’s probably where we get in a lot of physical activity input I suppose. I’d like to hear somebody stand up there and say, this is our policy that we’ve shown the governors and it’s been approved." P12  “I think policy for me is the big one. Because it’s always the hardest to address and even if we go in there with the aim of addressing policy, we end up addressing an opportunity.” P2 | Inner setting   - Culture |
|  | SLT Support of CAS Required to Leverage Whole-School Buy-in | “And dragging along my headteacher to come to the last conference and the conference before that really helped, because he was on board with it and wanted to action it. So, that was helpful” P18  “I just think it’s been slow moving in mine because I need to get SLT on board. I need the staff to buy-in, it would be helpful to have more time to try and implement it. I can see the benefits of it and I think it will be really good in our school and there’s a place for it. I just think next year it needs to be much more of a focus for us to push it forward.” P38  “I think what’s made the difference with those schools is that they’ve got someone on the management team, so like both deputy heads, I’ve met with the heads as well, the headteachers of both schools, so someone high up in the school is taking ownership over the project and I think that really makes a difference.” P9  “I think SLT are big drivers for any change in a primary school, I think they sometimes underestimate what a big impact they have across their school.” P7  “The whole of SLT is on board but upskilling teachers and explaining the reasons behind it, we’ve had 2 CPD sessions and I think that’s helped when we speak to people and explain the reasons, then you get them on board.” P25  Attending conferences: “…our heads need to be here [CAS conference], and our PE lead needs to be here to invest in it…” P32  “My SLT will sort of just say yes to stop me from bothering them. My deputy head came today, it’s the first one that any of the SLT have come to and there were a few ideas, I want to implement that and that and that so hopefully that will actually mean I have support rather than just appeasing me.” P21  “If the school doesn’t have SLT buy-in, they’re not going as quick.” P2  “And I think what’s made the difference with those schools is that they’ve got someone on the management team, so like both deputy heads, I’ve met with the heads as well, the headteachers of both schools, so someone high up in the school is taking ownership over the project and I think that really makes a difference.” P9  “I think that the most important thing for me, and I've learned over the year is changing senior leaders understanding of what CAS is about and it isn't about PE and sport” P1 | Inner setting   - Leadership engagement - Available resources   Process   - Opinion leaders |
|  | How CAS is Operationalised in School (Endemic Top-down School Approach) | "Again, this all landed on to me about October time. I became PE lead because the PE lead left so it was a lot to get my head round. The spring conference was the first conference I attended. B’s been helping me get my head around everything. " P25  "Same. It was the head that passed it over to P24 and I…" P22 | Inner setting   - Culture - Leadership engagement   Process characteristics   - Formally appointed internal implementation leaders |
|  | CAS Seen as Synonymous with PE & Sport | “I’ve felt like I’m the lone ranger, sort of thing, at my school, but I’m… They see me as PE and sport and physical activity or whatever that is, and they’ll do the rest.” P18  "Getting staff on board was a challenge because our PE lead/teacher left.” P25  “It’s creating more effective role models because I say, when I first started CAS, we’ve said in our first meeting, I’m literally the sole role model for physical activity in sport. I’m the only one, whereas since implementing this and sending people on the Move and Learn course, I can now safely say it’s not just me promoting physical activity and having that role model, so there’s someone now for the girls to look up to in our school instead of just me.” P19  Misconceptions that PA replaces PE: “I’ve struggled a lot with the demand to move away from PE towards physical activity, when I think they’re both actually equally valuable." P18 | Inner setting   - Compatibility - Relative priority |
|  | Challenges to Staff Buy-in | Competing priorities  But there’s so much else, so many other things to do, and maybe prioritise in the school that it’s hard sometimes to get it up the level of importance. That’s what I feel anyway" P13  "….Getting other teachers on board is a bigger part of it. But there’s so much else, so many other things to do, and maybe prioritise in the school that it’s hard sometimes to get it up the level of importance. That’s what I feel anyway" P13  Perceived lack of capacity/time  “The way our school is at the minute, probably the buy-in is a little bit harder to try and implement what we want to try and implement with timetabling, staffing.” P16  "I think I just need to make it clear to the teachers that it’s not necessarily extra work; it’s just a simple tweak on your lesson and it’s not like 50 burpees in the middle of Maths when they’re working out a problem-solving and reason, it’s just small things that you can change but then they’re then out of the sea and then they’re back in the sea, because I think some of mine are stuck in the ways of ‘I’m not doing that, it’s more planning, that’s extra work’, when actually it’s not.” P37  Lack of interest in PA  “They’re not active themselves. They’re not really having much interest in it” P8  Onboarding needed before changes can be made  “You need to get people on board otherwise things won’t happen, you can’t implement a new skill." P22  Struggling to make habitual changes:  "...But I am finding it challenging to get them to change the day-to-day routine, things like breaktimes and lunchtimes and getting kids active in call lessons and things like that…" P21 | Inner setting   - Relative priority - Compatibility   Individual characteristics   - Knowledge and beliefs of the intervention - Other personal attributes |
|  | Changes to Staffs Mindset Since Adopting CAS  -  *Initial Identified changes in school culture* | “It’s their mindsets and understanding of why physical activity is important, some of them didn’t really know much around it, that was a starter” P3  “PE hasn’t just been all about sport, it’s been about being physically active. I think it’s the terminology, getting staff used to terminology because when you say physical activity, they think It’s PE. " P25  "Then with staff training, the first session, that’s massive because the staff obviously, most staff don’t understand it, a bit like what happened this morning, like we’ll just come in and we’re doing circuit training, and all that. And the daily mile, and they do like the staff training, and when I did the staff training, I did some of those things that P12 did, with like moving around, and then they get it. But it’s not there, it’s nowhere near perfect, but the more that staff understand that it is for a health benefit.” *P11*  Purposeful decisions around spending (CAS/Pupil Premium) funding  “I feel like this year our spending of the PE and sport premium has been really good and I feel like we’re kind of making those small steps to becoming more active. So pretty positive.” P9  “Yes. I think it’s, like we were saying, the money was the big attraction, when actually, it’s really quite good that everyone’s sat on the pot of money, because everyone’s just willing to… I can see that we’re doing a lot of the work, but it all needs fine-tuning, and it needs tweaking a little bit.” P31 | Inner setting   - Culture   Characteristics of individuals   - Knowledge and beliefs about the intervention |
| Dose (satisfaction) | General Satisfaction with CAS | “Creating Active Schools has been fantastic.” P19  “I agree. I think CAS, the CAS framework is a fantastic tool for school development…. I think that’s brilliant…” P33  “The CAS programme so far has been quite supportive. There’s lot of things that they have on board with us and vice versa so we are getting the support where we need it so I’m very happy.” P38 | Intervention characteristics   - Relative advantage - Design quality and packaging   Inner setting   - Compatibility |
|  | CAS Champions and Facilitator | “And because he’s got the time to come out, he comes and helps us now.” P24  “the support we’ve had with [our CAS champion] has been good” P13  “Having somebody [in relation to the CAS champion] who’s not connected with the school is really important, you can sound off ideas to setup and using your experience and guides appropriately, and that’s what we have now.” P11  “I think it was to start because we were a bit like haven’t we heard from P2 for a while, have we heard from… and then like after Christmas I think…” (P35) … “Yeah, we realised we have to kind of [had to] reach out and push to get anything we wanted.” (P40)  “So, mine’s [CAS champion] changed. I was in a similar boat to you and then I haven’t had my staff meeting yet…” P19  “I don’t know that I’ve done that much work as a CAS champion overall…” P18  “I’m more gutted that P1’s leaving. Yes, I think he’s been at the centre of most school’s journeys and he’s there when JU:MP is overwhelming and the pressure you get. [They] just like, you don’t need to do that. That’ll be a huge loss.” P22  “Mine was quite good because I had a one-to-one session with P1. [They] spent two hours explaining everything to me. It was so straightforward, simple and it made sense to me. But, again, like I say, I wasn’t exposed to that first conference.” P25 | Process characteristics   - Champions - Engaging - External change agents |
|  | CAS Communities of Practice Facilitating Networking Opportunities | “Yes. The best thing for our schools is always this networking.” P18  “I think it’s also the time as well, so having the time to reflect at the conferences and a time to reflect at the meetings.” P9  “[The conferences are] brilliant days, and the information that people are giving you, great. Write loads down, but then after you leave here, it really is just down to you to take this away and go and implement it, isn’t it…” P16 | Intervention characteristics   - Relative advantage   Process characteristics   - Engaging - Executing - Reflecting and reviewing   Outer setting   - Cosmopolitanism |
|  | Administrative Tasks (e.g., profiling tool) Seen as Laborious But Generally Beneficial | “Yeah, definitely. [the profiling tool] It is a fantastic tool, it makes you think of all the areas.” P10  “But I think the actual toolkit at the beginning to identifying your needs was useful. admittedly, I haven’t looked at that since. But it would be interesting to do it again in September and see those differences.” P34  “The profiling tool is really good because it highlighted specific areas, maybe they’re not necessarily choosing which areas to prioritise so that’s really good.” P38  “So, we were sitting down and going through the hundred questions, and it just got so repetitive at the start, I can see why it were needed” P3 | Intervention characteristics   - Cost - Relative advantage   Process characteristics   - Reflecting and reviewing |
|  | JU:MP Related Satisfaction | “I'm not as convinced yet about the JU:MP stuff as I am with the CAS stuff.” P26  Fun days +/-  “We had some really successful JUMP days at our school which were really well picked up by parents and children. That was really good.” P21  “So for me the JU:MP day is a lovely day, as in they absolutely loved it, thought it was brilliant, but come the following day, nothing has changed.” P5  Neighbourhood meetings +/-  “I think the neighbourhood forums is a good way of networking, if you know who to get in contact with [the Active Travel lead] who has been able to rollout our CPD from scooter training, balance bike training, so that plays into it as well.” P14  “he neighbourhood there was lots of time just going over things that we’d been over before, and I think that’s particularly frustrating for a teacher or someone who works in a school, because I think sometimes when you don’t work in a school you don’t appreciate how literally you get in in the morning and it’s boom, boom, boom, all day just keeping you busy.” P9 | Outer setting   - Cosmopolitanism |
| Complexity | Multiple Health-based Projects in Bradford Causing Perplexity | “I think the first thing to say is and this is both a positive and a negative is we have to look at almost using CAS as the golden thread or the common thread, because you had so many different programmes going on you were already getting schools. And we still get this now. And actually, we haven't resolved it, which is a shame, which is schools go well, what's CAS what's JU:MP, what Living Well, and we just want one thing, and we tried to make that one thing, Creating Active Schools (CAS). But actually, there's been kickback on that, because the wider programmes want to make sure that their wider initiatives are being properly addressed” P1  “I still think that tension between schools and those programmes is going to continue until someone goes, what are we going to call this for Bradford? And yes, we might use Creating Active Schools as the underlying vehicle. But it almost needs to be right is this Living Well, and physical activity strand for Living Well, we use Creating Active School” P1  Initial confusion at CAS Conference  “So in feedback from the headteacher, she was a bit confused about how everything works together, so how CAS and how JU:MP work together. From my perspective, I don’t think it really… I personally don’t think it really matters, it’s all under the umbrella…” P9  Specific lack of clarity for CP5 schools  “I just think it’s for CP5, it’s actually what is it? Is it still running, and what are the next steps if it is, for me.” P16  “Our CP5 money has been difficult to get hold of because I didn’t fully understand who we were meant to be emailing to get the money from, my business manager wasn’t particularly capable of doing it or sending it off, because we’re an academy and there’s a finance group you’ve got to… so I’m still not actually sure if we’ve got the £4,000 yet that we’re meant to be getting as a CP5 school.” P7 | Outer setting   - Cosmopolitanism - Peer pressure   Intervention characteristics   - Intervention source |
|  | Initial Bewilderment Alleviated Over Time | “Yes, it made me feel like I was a bit of a plonker, that I was the only one in that room who didn’t have a clue, either what this was or what on earth you were all talking about. Then, when we got to the second one [conference], we were like, do you have a clue? I have no idea!” P23  “I sat on the wrong table. It was a nightmare but they were all really lovely on that day as well. I can’t say anything bad about them. Because it’s their baby, they’re so passionate about it. It was just boom, boom, boom.” P22  “I think, for me, there was some confusion at the start, like you said, there’s different elements to it [the CAS framework], and I assumed it was all in one pouch.” P32  For those that got involved post planning there was less confusion:  “I think because I didn’t have that first conference with all that information thrown at me, I found the planning bit a bit easier” P21 | Intervention characteristics   - Complexity |
|  | CAS Champion Support Increasing Clarity for School Staff | “This person is so key to making sure that your schools are not lost in this fog of, like, everything.” P31  “I just want to say that I wouldn’t have been able to do it without P7, my CAS champion with me. I’d still be just looking at this, so confused. He’s been really helpful.” P21 | Intervention characteristics   - Complexity   Process characteristics   - Champion   Engaging |
| Self-efficacy | Development of School Staff’s Confidence | " I think it’s the training, the staff are scared. I think they think, this is too overwhelming, you want me to go outdoors, you want me to stop what I’m supposed to be doing and so something completely different and be more active and how am I supposed to do that? I think it’s a lot of training and just educating them that this is a positive thing, it’s not adding to workload, it might actually make it a little bit easier." P22  “And they’re [behaviour concerns] all genuine fears I think as well, they’re all genuine barriers” P39  Training benefited confidence levels:  “One of the Year 3 teachers, she said, oh it was just brilliant, like the training and then to give her the confidence to do it” P13 | Individual characteristics   - Knowledge and beliefs of the intervention - Self-efficacy   Inner setting   - Access to knowledge and information |
|  | CAS Champions Differing Capabilities | “I didn’t feel like I was capable to sit in a room with a school leader and, you know, people talk about the imposter syndrome, we’ve referenced it loads of times, but sometimes I don’t think it’s imposter syndrome, for me, I think it’s respect for school leaders like educated people. I don’t want to be forcing an agenda on them” P6  “I would say to do the profiling tool you need that joint. And it needs somebody to lead the questions as well. Because I’m not a teacher and I came from nutrition, I did not like understand the depth that the questions could reach. Whereas [XX Champion], being a teacher and involved in the making of the profile, the framework, consultation phase, [they] really got all the questions and understood how deep schools needed to answer each one.” P2  “I think somebody who’s in the role, who has a little bit of background as a collective to offer physical education, are probably a little bit more prepared for that level of vigorous paperwork but then the more you did a bit deeper, to use that phrase again, I think the CAS leads in the schools are all very different” P6  “I've felt like I don’t know what I’m doing quite a lot of the time with it based on the amount of time I’m able to commit to doing the CAS Champion role based around things at school.” P7 | Process characteristics   - Champions   Intervention characteristics   - Design quality and packaging   Individual characteristics   - Self-efficacy |
| Perceived effectiveness | Positive Changes to School Policy | “I’ve never pestered my heads or pushed enough for the school to change its policies, and the school’s policies have changed this year. So, we got written into our school improvement plan that we would look at physical activity and the physical activity had to improve.” P18  “It’s in our CPD policy and it’s on the school development plan, and part of our plan next year is to make sure that it’s sort of prevalent in everything that we communicate to parents as well…” P35 | Inner setting   - Culture - Implementation climate   Process   - Executing |
|  | Positive Changes to the School Environment | “And by working with the staff as stakeholders, we’ve then been able to address the environment. So, for example, one of our schools has just a concrete playground. But because we worked with lunchtime staff there, we empowered them, we encouraged the play at lunchtimes, they’ve now got £50k worth of outdoor grants to build up their outdoor space” P2  “They’ve used some of the developers that we used at our school to look at their green space because they don’t have a great deal in the school grounds but they’ve got a separate piece of green space around the corner from the school. So a development has already started happening. They’re looking at collective solutions to combine with schools in their local community to appoint… that’s not just funding from CAS, I think that’s a little bit from Premium and maybe some from the trust that they’re now part of to sort of all put into a pot to appoint a forest school leader to utilise the green space for the schools in the community.” P6  “I think maybe we're having a rethink about what we might use that money for. We were additionally thinking about running track. I think maybe minds are changing” P30 | Inner setting   - Culture - Structural characteristics - Implementation climate   Process   - Executing |
|  | Positive Changes to School-based Stakeholders | “~~The biggest thing here is the whole school staff training is a slow shift in mindset of people in schools around the perceptions on physical activity, school sport and PE. And that wider framework that CAS is a commitment and a willingness to embrace it and a starting of some interventions that are having minor impact on physical activity behaviours” P1~~  “So, staff empowerment has been massive through CAS. And I would say that that’s been one of the biggest wins in the school so far.” P2  Intended Outcomes (Networking) of Conferences & Communities of Practice  “I think the networking. The points of contact. Knowing that there’s someone there to support you in that process.” P34  “I, like, listened to the different cases than this and then I think you can think that might work in my school or a version of that might work, or I might pitch that idea, or we could maybe adopt that idea. So, yeah, the shared stuff is good stuff.” P26  Also good to share ideas for the champions: “It’s been a really positive experience; it’s been good to meet with other people, particularly the peers that you get to speak to, sharing ideas with the other CAS Champions.” P7  “We wouldn’t have been able to deliver that if it weren’t for each other supporting each other” P34  “We went up to the academy in [a CAS school and] watched them do their Active Enrichment and it’s something that we implemented fairly quickly after our first visit. We got everything up and running so that’s been going for a couple of months. That’s working really well. We’ve also joined one of, it’s not a toolkit, I’m not sure what you call it as we’re also a RIC school and a JUMP school as well.” P23 | Inner setting   - Culture - Networks and communications   Outer setting   - Cosmopolitanism   Process   - Executing |
|  | Increases in PA Opportunities | “After we had some CPD from our CAS champions, one of the teachers in my school started an afternoon breaktime, which we don’t normally do.” P21  “We’ve been working on our Active Enrichment. That’s one thing we have implemented along our journey so far,” P23  “Yeah, so we’re focussing really on the opportunities now because we’ve got the active travel as well, we’ve got the Wild Tracker like I mentioned earlier, and then we did have [X Champion] and P2 coming to teach the play leaders, buddies, and lunchtime supervisors on what we could it at breaks to make…” P40  “I’ve used some of the money we’ve had to start afterschool clubs for the children, they all really want to do it. I’ve gone out of my way to make it become something they haven’t done before so it’s not football or cricket, it’s archery and nature and Jujitsu and fencing, sports they don’t normally do and they’re really into that.” P21 | Inner setting   - Implementation climate - Culture   Process   - Executing |
|  | Perceived Impact on Children’s PA Levels | “the Moki bands, which are a band which basically records a child’s amount of steps that they do and it collates all that data. So at the beginning of the year they did a baseline assessment, which I think was further motivation for them because they saw that actually even their sporty children, what they would consider their most active children, weren’t getting the recommended 30 minutes provided within school. So they just did a really simple thing, they just, as part of our staff workshop they had some time then to think about how to make PE lessons more active, because it was something that quite shocking for them was that PE days, they’ve got a whole afternoon of PE and they’re still not getting 30 minutes of physical activity – how is this happening? So they literally just focused on PE and they got the data… I couldn’t quote the data now, but there was an increase, more so for boys, but for both boys and girls, and their physical activity has improved and PE is now a more active less.” P9  “So, from all the things that we’ve just said about having scooters and bikes and things in school, we’ve started to use our scooters and bikes as movement breaks from some children that are on alternative timetables. So, if they need a movement break in the morning or the afternoon, they have a 15-minute timetable session where everyone else is inside and they go out with their support and then they access the scooters or the bikes, and it’s been amazing. You can see them just bubbling and they just need that change of environment and change of face and so they’ve just been going out on the bikes, having five minutes, and it just kind of resets them, so I think the teachers and the staff in school, we don’t really know, because you’re just in it, but when you take a step back, you think, that has been such a good way of managing behaviour…” P31  “if you did that ripple effect of actually what you have done and what has changed, is probably a lot more than what you think, but I think if you know you could do so much more…” P37 | Process characteristics   - Executing |
| Recommendations for Future Development of CAS | Opportunity for Networking | “Although we’re networking today, it’s very guided activities, but an opportunity in local clusters say by led by you, for all of the CAS schools, just the leaders, just to get together and brainstorm and thrash and problem solve” P32  “I was looking at CAS’s twitter like there’s nothing on here at all. So if you just stick a video on, it’s literally a mini toolkit early years” P4 | Outer setting   - Cosmopolitanism   Process   - Engaging |
|  | CAS Programme still in Development, with Suggested Evolvements Over Time | “Start to rank those schools. And then to start to look at what that what does that mean for the way we support them moving forward, because actually, some might benefit from going back to the toolkit and doing online CPD, whereas others might need more intensive, CAS champions support, we need to be more flexible about the ways we use those types champions, because some of them are really skilled at going into schools and helping them set up, you know, outdoor learning initiatives, whereas others are better at the more sort of advice and signposting sort of support. So it's about how we use the different strengths we have in the team.” P1  “My view is, in future, you might not have a full day of a conference, you might have half day of a conference, and then a half a day of a school visit, where you just go, right, any schools that want they're interested in outdoor learning, we've got you might say, we've got three different site visits . On this day, one's outdoor learning, one's active learning, one's active travel, sign up to the ones you want to go to.” P1  “I think that's the bit I think I'd like to see change over time is either how do we better release the CAS champions or how do we work with schools so that CAS champions get this dedicated release time to help other schools and then how do we make them better quality” P1 | Intervention characteristics   - Adaptability - Trialability |
|  | Leveraging School buy-in Through Award Recognition Scheme | “…talking about the Yorkshire Sports Centre of Excellence Award, like if you get that for your school, there’s a lot of credibility and lots to celebrate within your school and your Trust even wider, so it’s almost trying to get the attention of school leaders or perhaps even beyond that, your Academy Trust, and can it get the attention, look at this that we’ve managed to achieve, and again I keep talking about that hook. You need that initial hook in.” P6 | Outer setting   - External policies and incentives |
